# Efficacy of Dolutegravir versus Darunavir in Antiretroviral First-Line Regimens According to Resistance Mutations and Viral Subtype

**DOI:** 10.3390/v15030762

**Published:** 2023-03-16

**Authors:** Pierluigi Francesco Salvo, Damiano Farinacci, Arturo Ciccullo, Vanni Borghi, Stefano Rusconi, Annalisa Saracino, William Gennari, Bianca Bruzzone, Ilaria Vicenti, Annapaola Callegaro, Antonio Di Biagio, Maurizio Zazzi, Simona Di Giambenedetto, Alberto Borghetti

**Affiliations:** 1 Dipartimento di Sicurezza e Bioetica, Sezione di Malattie Infettive, Università Cattolica del Sacro Cuore, 00168 Rome, Italy; 2 UOC Medicina Protetta–Malattie Infettive–ASL Viterbo, 0100 Viterbo, Italy; 3 Malattie Infettive, Ospedale San Salvatore, 67100 L’Aquila, Italy; 4 Clinica delle Malattie Infettive e Tropicali dell’Università di Modena e Reggio Emilia, 41100 Modena, Italy; 5 UOC Malattie Infettive, Ospedale Civile di Legnano, ASST Ovest Milanese, 20025 Legnano, Italy; 6 Clinica Malattie Infettive, Università degli Studi di Bari, 70121 Bari, Italy; 7 Hygiene Unit, Policlinico San Martino Hospital, 16126 Genoa, Italy; 8 Department of Medical Biotechnologies, University of Siena, 53100 Siena, Italy; 9 Microbiology and Virology Unit, ASST Papa Giovanni XXIII, 24127 Bergamo, Italy; 10 Infectious Diseases Clinic, Policlinico San Martino Hospital, Department of Health Sciences (DISSAL), University of Genoa, 16126 Genoa, Italy; 11 UOC Malattie Infettive, Fondazione Policlinico Universitario A.Gemelli IRCCS, 00168 Rome, Italy

**Keywords:** HIV drug resistance, HIV genotypic resistance testing, HIV viral subtype, antiretroviral therapy

## Abstract

Background: Dolutegravir (DTG)-based first-line regimens have shown superior efficacy versus darunavir (DRV)-based ones in randomized trials. We compared these two strategies in clinical practice, particularly considering the role of pre-treatment drug resistance mutations (DRMs) and of the HIV-1 subtype. Materials and methods: The multicenter Antiretroviral Resistance Cohort Analysis (ARCA) database was queried to identify HIV-1-positive patients starting a first-line therapy with 2NRTIs plus either DTG or DRV between 2013 and 2019. Only adult (≥18 years) patients with a genotypic resistance test (GRT) prior to therapy and with HIV-1 RNA ≥1000 copies/mL were selected. Through multivariable Cox regressions, we compared DTG- versus DRV-based regimens in the time to virological failure (VF) stratifying for pre-treatment DRMs and the viral subtype. Results: A total of 649 patients was enrolled, with 359 (55.3%) and 290 (44.7) starting DRV and DTG, respectively. In 11 months of median follow-up time, there were 41 VFs (8.4 in 100 patient-years follow-up, PYFU) and 15 VFs (5.3 per 100 PYFU) in the DRV and DTG groups, respectively. Compared with a fully active DTG-based regimen, the risk of VF was higher with DRV (aHR 2.33; *p* = 0.016), and with DTG-based regimens with pre-treatment DRMs to the backbone (aHR 17.27; *p* = 0.001), after adjusting for age, gender, baseline CD4 count and HIV-RNA, concurrent AIDS-defining event and months since HIV diagnosis. Compared with patients harboring a B viral subtype and treated with a DTG-based regimen, patients on DRV had an increased risk of VF, both in subtype B (aHR 3.35; *p* = 0.011), C (aHR 8.10; *p* = 0.005), CRF02-AG (aHR 5.59; *p* = 0.006) and G (aHR 13.90; *p* < 0.001); DTG also demonstrated a reduced efficacy in subtypes C (versus B, aHR 10.24; *p* = 0.035) and CRF01-AE (versus B; aHR 10.65; *p* = 0.035). Higher baseline HIV-RNA and a longer time since HIV diagnosis also predicted VF. Conclusions: In line with randomized trials, DTG-based first-line regimens showed an overall superior efficacy compared with DRV-based regimens. GRT may still play a role in identifying patients more at risk of VF and in guiding the choice of an antiretroviral backbone.

## 1. Introduction

The latest European HIV treatment guidelines for drug-naive persons living with HIV (PLWH) recommend the use of two nucleoside reverse transcriptase inhibitors (NRTIs) associated with an integrase strand transfer inhibitor (InSTI) as first-line regimens for most patients. Non-nucleoside reverse transcriptase inhibitor (NNRTI)-based regimens are also considered as alternative regimens in specific clinical situations, as well as darunavir (DRV)-based regimens [1].

INSTIs are effective and well tolerated molecules [2]. DTG, in particular, is an integrase inhibitor approved for once-daily dosing without pharmacokinetic boosters in naive PLWH, often preferred as a first-line treatment because of its antiviral efficacy, high genetic barrier and limited drug–drug interactions [3].

Boosted protease inhibitors, and particularly DRV, have also demonstrated great virological effectiveness in clinical trials and in clinical practice [4], and the development of resistance-associated mutations with their use is even less frequent than with DTG [5]. Despite that, the need for pharmacological boosting leading to clinically important drug–drug interactions, as well as concerns for cardiovascular and metabolic issues [6], has reduced their clinical use in more recent years.

A GRT is recommended prior to the initiation of antiretroviral therapy (ART), ideally at the time of diagnosis [1]. However, in clinical practice, this is still a challenging goal to reach in many clinical settings, even in the most advanced ones, due to the reallocation of laboratory resources after the COVID-19 pandemic. Therefore, it is not unusual to start treatment without information about drug resistance mutations (DRMs) or about the viral subtype, two factors that can impact the virological response [7,8,9,10,11].

Different studies compared DTG- to DRV-based regimens as first-line treatments for adult naive PLWH [12,13]. In FLAMINGO, once-daily DTG-based regimens were associated with a higher virological response rate than once-daily ritonavir-boosted DRV-based ones [12]. Conversely, a more recent prospective, multicenter, non-inferiority trial performed in Kenya, Uganda and Zimbabwe, analyzing the efficacy of DTG-based regimens versus DRV-based ones after a first-line treatment failure [13], showed a comparable effectiveness of DTG and DRV for the outcome of viral suppression, underlining the good efficacy of the two anchor drugs in the setting of DRMs. This trial also showed the occurrence of major DTG resistance mutations in the subgroup of patients with a confirmed VF, while no major DRV resistances have been found.

Regarding pre-treatment DRMs and their possible influence on time to virological failure, Kityo et al. [10] analyzed the clinical and virological development of children aged <12 years who initiated first-line ART in Uganda during 2010 and 2011, collecting data regarding viral load and GRT at baseline and in cases of VF. Results from this study showed that in the group of children who experienced VF, the presence of pre-treatment DRMs was the strongest predictor of VF and acquired DRMs.

Hamers et al. [11] similarly analyzed the effect of pre-treatment DRMs on the response to first-line ART in a multicenter cohort study conducted in sub-Saharan Africa. They enrolled a total of 2733 PLWH, starting a NNRTI-based regimen from 2007 to 2009. Compared with participants without a pre-treatment of DRMs, the odds ratio for VF and acquired DRMs was increased in participants with pre-treatment DRMs to at least one prescribed drug.

Concerning viral subtypes, different studies have tried to analyze the possible influence of the HIV-1 viral subtype on virological responses to antiretroviral therapies, with sometimes discordant results [9,14,15,16,17,18,19]. Haggblom et al. [9] analyzed the virological response of a cohort of 1077 PLWH in Sweden, focusing on the differences between the group of PLWH harboring a B subtype and the group of PLWH with a C subtype infection. The findings from this study suggest an increased risk of virological failure in PLWH harboring a C subtype, especially in those treated with a boosted protease inhibitor-based regimen. Conversely, another study conducted in the United Kingdom [19] on the virological effectiveness of antiretroviral regimens containing tenofovir noticed no differences between B and non-B subtypes, suggesting that there is no intrinsic effect of viral subtype on the efficacy of antiretroviral regimens containing tenofovir.

We undertook this study to assess how DTG-based regimens perform compared to DRV-based ones in the treatment of naive PLWH in clinical practice, focusing on the possible influence of pre-treatment drug resistance mutations and viral subtypes on the time to virological failure.

## 2. Materials and Methods

### 2.1. Study Population

A retrospective study was performed using the Antiretroviral Resistance Cohort Analysis (ARCA) database [20], which contains data on ART and HIV resistance from more than 40,000 patients in Italy. At the time of writing, all the sequences available were generated by Sanger sequencing, based on commercial systems or in-house protocols. Patients were selected from among those starting a first-line DTG- or DRV-based regimen from January 2013 to December 2019.

Patients were considered eligible for the study if they had a concentration of plasmatic HIV-1 RNA at a baseline of 1000 copies/mL or higher, with no previous exposure to antiretroviral treatments and with a genotypic resistance test available prior to starting ART. Major NRTI, NNRTI, PI resistance mutations were identified using the 2019 IAS-USA drug resistance mutation list [21]. Importantly, GRTs for INSTIs were available only for a minority of patients who started on DTG.

### 2.2. Objective

The primary outcome was to compare three-drug regimens with 2NRTIs plus DTG versus 2NRTIs plus DRV in the time to virological failure (VF), defined as the detection of an HIV-1 RNA of 50 copies/mL or higher after at least three months from the start of the therapy, focusing on the presence of pre-treatment drug resistance mutations (DRMs) and the different viral subtypes.

We considered as DRMs those mutations that could cause at least potential low-level resistance to one or both of the NRTIs included in the study regimen, by using the Stanford HIVdb genotypic resistance interpretation system (version 9) [22].

### 2.3. Statistics

Baseline patients’ characteristics were described as numbers and proportions for qualitative variables and medians and interquartile ranges (IQR) for continuous variables. Differences in baseline characteristics between DTG- and DRV-based regimens were assessed through chi-squared testing for qualitative variables and Student’s t-testing for continuous variables. Estimated probabilities of VF were calculated for both groups through the Kaplan–Meier estimator and compared through the log-rank test. Two multivariable Cox regression models were then performed to detect any differences between DTG and DRV in time to VF: in the first model, the exposure (ART group) was stratified for the presence or absence of any pre-treatment DRMs associated with at least potential low-level resistance to at least one of the 2 molecules of the backbone and adjusted for viral subtype and for any potential confounder; in the second model, the effect of the ART regimen was analyzed by stratifying the ART group for viral subtype (B versus every other subtype) and by adjusting for pre-treatment DRMs (presence versus absence of at least one potential low-level DRM to the backbone) and the same pattern of confounders.

A confounder was defined as any demographic and viro-immunological variable that resulted in a statistically different value at baseline between the two ART groups and that could affect treatment outcomes, according to previous knowledge [23,24,25,26,27]. In particular for this study, the confounders that we found to be statistically significant were age, gender, CDC stage at baseline, time since HIV diagnosis, HIV-1 viral load at baseline and CD4 cell count at baseline.

## 3. Results

A total number of 649 patients was eligible for study analysis. Among them, 359 (55.3%) started a DRV-based regimen and 290 (44.7%), a DTG-based one. Dosing of the DRV and type of booster was not reported in the electronic database.

The sociodemographic and viro-immunological characteristics of the enrolled patients are listed in Table 1. Most subjects were men (76.9% in the DRV group and 75.9% in the DTG group), with a median age of 41 years old. The DRV group differed from the DTG group for a slightly older mean age, a longer time since HIV diagnosis, a higher frequency of current AIDS-defining events, a lower baseline CD4 cell count and a higher baseline plasmatic viral load. In both groups, patients were more often infected with an HIV-1 B subtype; however, 31.4% of the population harbored a non-B subtype.

Concerning the pre-treatment DRMs, 30 patients (4.6%) had a virus with at least one NRTI-associated potential low-level resistance mutation (18 and 12 in DRV and DTG groups, respectively); in nine cases (1.4%), pre-treatment DRMs were associated with at least potential low-level resistance to the backbone (four and five in the DRV and DTG groups, respectively). All the mutations of the population are listed in Table 2. The most commonly detected pre-treatment DRMs conferring at least potential low-level resistance to one or both NRTIs employed in the regimen were: M184V and D67N (four cases each) and L210W and M41L (three cases each). M41L was always combined with other DRMs, contributing to a reduced susceptibility to both abacavir and tenofovir. M184V is known to cause high-level in vitro resistance to 3TC and FTC and low/intermediate resistance to ABC. D67N and L210W usually do not cause resistance to the NRTIs investigated in the present study, but they were always associated with other mutations.

Pre-treatment DRMs were associated with VF in two patients on tenofovir/emtricitabine plus dolutegravir, one of whom presented only an M184V mutation and the other showing a combination of M41L and T215C/F/I/L/R/S (particularly, T215I can increase the resistance mutation score of tenofovir, as reported by the Stanford interpretation system [23]; this mixture of mutations also usually arises from viruses that once contained T215Y/F, which in turn can contribute to reduced abacavir and tenofovir susceptibility in combination with M41L). The patterns of pre-treatment DRMs conferring resistance to the study backbones are summarized in Supplementary Table S1.

In one patient in the DRV group, the combination of D30N, M46L and I54L mutations was detected, conferring low-level resistance to DRV. Similarly, in one of 121 patients in the DTG group with an available pre-treatment GRT to InSTIs, intermediate resistance to the anchor drug was revealed, due to the presence of the R263K mutation. Importantly, resistance to the anchor drug was neither associated with resistance mutations to the backbone nor to VF in both groups.

During 11 months of median follow-up time, 56 VFs occurred in the overall population: 41 in 488.53 patient-years of follow-up (PYFU) in the DRV group (incidence rate of 8.4 in 100 PYFU) and 15 in 285.7 PYFU in the DTG one (incidence rate of 5.3 in 100 PYFU). The estimated probabilities of VF for the DRV group were 10.9% (95% confidence interval (CI), 7.7–15.5%) and 16.7% (95% CI, 12.2–22.6%) at 1 and 2 years, respectively. For the DTG group the estimated probabilities of VF were 4.8% (95% CI, 2.5–9.0%) at 1 year and 11.9% (95% CI, 6.9–20.3%) at 2 years, resulting in a borderline significant log-rank for DTG versus DRV (*p* = 0.067).

After adjusting for gender, age, baseline CD4 cells count, zenith HIV HIV-RNA, months since HIV diagnosis and CDC stage C (Cox model 1), starting a regimen with 2 fully-active NRTIs plus DRV was associated with a higher risk of VF if compared with patients starting a DTG-based regimen with a fully-active backbone (adjusted hazard ratio (aHR), 2.33; 95% CI, 1.17–4.66; *p* = 0.016), especially in patients with a history of HIV infection longer than 1 month or a viral load at baseline higher than 100,000 copies/mL. Moreover, DTG-based regimens with a compromised backbone (i.e., a virus with at least low-level resistance to one of the two NRTIs) were at higher risk of VF compared with DTG plus a fully active backbone (aHR 17.27; 95% CI, 3.24–92.15; *p* = 0.001). Since patients on DRV-based regimens with pre-treatment DRMs to the backbone had no VF, no hazard ratios could be calculated for this group. Interestingly, among viral subtypes, the C subtype (compared with the B subtype; aHR 3.10; 95% CI, 1.07–8.98; *p* = 0.037) and G subtype (compared with the B subtype; aHR 4.23; 95% CI, 1.44–12.47; *p* = 0.009) showed an independently increased risk of VF. Table 3 reports the results of the first multivariable model.

In the second multivariable model (see Table 4), the ART group was stratified according to viral subtype (B versus any other subtype). Compared with patients harboring a B viral subtype and treated with a DTG-based regimen, patients on a DRV-based regimen had an increased risk of VF, both in B subtype (aHR 3.35; 95% CI, 1.33–8.44; *p* = 0.011), C subtype (aHR 8.10; 95% CI, 1.90–34.48; *p* = 0.005), CRF02-AG subtype (aHR 5.59; 95% CI, 1.62–19.24; *p* = 0.006) and G subtype (aHR 13.90; 95% CI, 3.30–58.57; *p* < 0.001). However, DTG also demonstrated a reduced efficacy in C subtype (versus DTG in B subtype; aHR 10.24; 95% CI, 1.12–93.55; *p* = 0.035) as well as in the CRF01-AE subtype (versus DTG in the B subtype; aHR 10.65; 95% CI, 1.18–96.04; *p* = 0.035). Direct comparisons of DRV and DTG for every non-B subtype did not show any difference in effectiveness between the two treatment groups. Particularly, no differences in VF were evidenced when directly comparing DRV-based to DTG-based regimens in the C subtype (DRV versus DTG; aHR 0.79; 95% CI, 0.07–8.54; *p* = 0.847).

## 4. Discussion

This study documents that DTG-based regimens are associated with better virological outcomes than DRV-based regimens in naive PLWH with fully active backbones and harboring a B viral subtype.

In the subgroup of patients with a fully active backbone, DTG showed higher rates of virological suppression compared to DRV, with a lower incidence of VF events. However, the efficacy of DTG was affected by the presence of mutations decreasing the activity of the NRTI backbone. It must be noted that all the HIV sequences considered were obtained by population sequencing; thus, the dataset may have included cases with undetected minority drug-resistant species potentially, impacting the response to therapy. It is indeed known that drug resistance mutations are fixed at different levels depending on the balance between the advantage and fitness cost [28], with the latter tending to prevail in the absence of therapy, such as in the pre-treatment samples analyzed in this study.

Regarding viral subtypes, PLWH infected by a B viral subtype in antiretroviral therapy with DTG-based regimens showed better outcomes in achieving and maintaining virological suppression than patients in DRV-based regimens with the B, C, CRF02-AG or G viral subtypes or patients on DTG-based regimens infected by the C or CRF01-AE viral subtype. Conversely, no statistical differences were found for DRV versus DTG for non-B viral subtypes.

These results seem to be in line with the ones of the FLAMINGO study [12], which showed a superior efficacy of DTG versus DRV/r in terms of time to VF. One of the differences between our work and the FLAMINGO study is that only patients with no resistance at screening were enrolled in the FLAMINGO trial. The efficacy of an antiretroviral regimen surely depends on different aspects, and the presence or absence of viral mutations linked to resistances to antiretroviral agents surely is a relevant variable to take into consideration. Until now, in clinical trials, the virological efficacy of DTG and DRV-based antiretroviral regimens has been analyzed in time to virological failure in patients with no particular viral resistance profiles. In this study, we aimed to analyze the efficacy of these antiretroviral regimens, focusing on the influence that the presence of pre-treatment viral mutations conferring resistance to drugs used by the patient could have.

An important trial comparing DTG and DRV in a more complex population, the NADIA trial [13], showed a non-inferiority of DTG- compared to DRV-based regimens as a second-line treatment in terms of virological suppression, but also showed an increased risk of development of mutations linked to the use of DTG compared to DRV in the presence of pre-existing NRTI mutations. In the NADIA trial, however, the study population was made up of PLWH with confirmed HIV first-line treatment failure, while we analyzed the virological response to study regimens in a population of naive PLWH with no previous exposure to an antiretroviral regimen. While taking into consideration the differences in terms of eligibility criteria for the NADIA trial and our retrospective study, in our population, a decreased efficacy of DTG if used in the presence of pre-treatment DRMs to the NRTI backbone employed could be seen. Even if no direct comparison could be performed for DTG and DRV in populations with pre-treatment DRMs, it is interesting to note that no VF occurred in the subgroup of patients on DRV and a compromised NRTI backbone. Altogether, these findings raise some concern about a more relevant effect of pre-treatment DRMs on DTG-based regimens than on DRV-based ones, in terms of viral suppression and the subsequent development of mutations.

The cohort of PLWH analyzed in this study is quite varied because it comes from the clinical experience of several Italian clinical centers, which provided data to the ARCA database. Clearly, each center had its own population and approach to treatment and this ensured a wide heterogeneity in the study population and therefore a good generalizability of the study results, at least among European countries. Conversely, a major limitation lies in the demographic characteristics of the population, mostly made up of men and Caucasians, making it difficult to generalize the results to other populations. Our study surely presents other limitations, including a lack of information about the presence of a possible booster (ritonavir or cobicistat) used with DRV and possible unknown confounders between the two studied populations. However, after stratifying for all measurable baseline confounders such as viral load, CD4 cell count and disease duration, a more favorable outcome with DTG was confirmed, suggesting a good reliability of our findings. Finally, no data regarding adherence to antiretroviral treatment were reported in the database. This surely represents a significant limitation, considering that a low level of adherence to treatment could affect virological responses and facilitate the development of DRMs.

The strengths of our work are the large sample size as well as the availability of real-life data coming from clinical practice and not available from the analysis of clinical trials.

In conclusion, from this study, DTG seems to be the best choice for clinicians facing naive people living with HIV starting a first-line ART. However, a possible reduced efficacy of DTG-based regimens is still possible in certain settings and the availability of GRTs prior to the beginning of a first-line antiretroviral regimen is of paramount importance in order to correctly weigh the individual risk of VF.

## Figures and Tables

**Table 1:** Characteristics of the study population, overall and separately, according to the treatment.

Variables	Total n = 649 (100%)	DRV Group n = 359 (55.3%)	DTG Group n = 290 (44.7%)	*p*
**Gender (n, %)**				**0.025**
- **Male**	496 (76.4%)	276 (76.9%)	220 (75.9%)	
- **Female**	136 (21.0%)	79 (22.0%)	57 (19.6%)	
- **Unknown**	17 (2.6%)	4 (1.1%)	13 (4.5%)	
**Age (years) (mean, SD)**	41 (±11)	42 (±11)	40 (±12)	0.066
**Ethnicity (n, %)**				**<0.001**
- **Caucasian**	343 (52.8%)	240 (66.9%)	103 (35.5%)	
- **African**	43 (6.6%)	24 (6.7%)	19 (6.5%)	
- **South American**	26 (4%)	12 (3.3%)	14 (4.8%)	
- **Other/Unknown**	237 (36.5%)	83 (23.1%)	154 (53.1%)	
**Risk factor for HIV (n,%)**				**<0.001**
- **Heterosexual**	145 (22.3%)	95 (26.5%)	50 (17.2%)	
- **MSM**	182 (28.0%)	130 (36.2%)	52 (17.9%)	
- **IDU**	19 (2.9%)	16 (4.5%)	3 (1.0%)	
- **Other/Unknown**	303 (46.7%)	118 (32.9%)	185 (63.8%)	
**CDC stage C at baseline (n, %)**	28 (4.3%)	23 (6.4%)	5 (1.7%)	**0.004**
**Baseline CD4 count (cells/mmc) (n, %)**				**<0.001**
- **≤200**	230 (35.4%)	149 (41.5%)	81 (27.9%)	
- **>200**	315 (48.6%)	174 (48.5%)	141 (48.6%)	
- **Unknown**	104 (16%)	36 (10.0%)	68 (23.4%)	
**Baseline CD4/CD8 ratio (mean, SD)**	0.37 (±0.32)	0.32 (±0.29)	0.44 (±0.36)	**<0.001**
**Baseline HIV-1 viral load (copies/mL) (n, %)**				**<0.001**
- **≤100,000**	279 (43.0%)	153 (42.6%)	126 (43.4%)	
- **100,000–499,999**	169 (26.0%)	113 (31.5%)	56 (19.3%)	
- **≥500,000**	86 (13.3%)	52 (14.5%)	34 (11.7%)	
- **Unknown**	115 (17.7%)	41 (11.4%)	74 (25.5%)	
**Time since HIV diagnosis (n, %)**				**<0.001**
- **≤15 days**	88 (13.5%)	60 (16.7%)	28 (9.7%)	
- **16 days–3 months**	153 (23.6%)	99 (27.6%)	54 (18.6%)	
- **3–12 months**	33 (5.1%)	24 (6.7%)	9 (3.1%)	
- **≥12 months**	86 (13.3%)	63 (17.5%)	23 (7.9%)	
- **Unknown**	289 (44.5%)	113 (31.5%)	176 (60.7%)	
**Backbone (n,%)**				**<0.001**
- **TAF (or TDF)/FTC**	525 (81.0%)	308 (85.8%)	217 (75.2%)	
- **ABC/3TC**	124 (19%)	51 (14.2%)	73 (24.8%)	
**HIV viral subtype (n,%)**				0.124
- **B**	445 (68.6%)	258 (71.9%)	187 (64.5%)	
- **A**	34 (5.2%)	22 (6.1%)	12 (4.1%)	
- **C**	24 (3.7%)	13 (3.6%)	11 (3.8%)	
- **CRF02_AG**	57 (8.8%)	28 (7.8%)	29 (10.0%)	
- **CRF01_AE**	12 (1.9%)	6 (1.7%)	6 (2.1%)	
- **F**	35 (5.4%)	18 (5.0%)	17 (5.9%)	
- **G**	23 (3.5%)	10 (2.8%)	13 (4.5%)	
- **Other**	19 (2.9%)	4 (1.1%)	15 (5.1%)	

TAF = Tenofovir Alafenamide Fumarate; TDF = Tenofovir Disoproxil Fumarate; ABC = Abacavir; FTC = Emtricitabine; 3TC = Lamivudine.

**Table 2:** Pre-treatment DRMs (n) observed in the study population, overall and separately, according to the treatment.

Pre-Treatment DRMs	Total n = 649	DTG Group n = 359	DRV Group n = 290
**TAMs**			
**D67N**	3	1	2
**D67N/S**	1	1	0
**D67D/N**	2	1	1
**M41L**	6	4	2
**M41M/L**	2	1	1
**M41M/I/L**	1	0	1
**L210W**	3	2	1
**K219E**	1	0	1
**K219Q**	3	1	2
**K70R**	1	0	1
**T215C/F/I/L/R/S**	1	1	0
**Non-TAMs**			
**M184V**	4	1	3
**A62V** **A62A/V**	2 1	2 1	0 0
**V75M**	1	1	0
**F77F/L** **F77S**	1 1	1 0	0 1
**K70K/T**	1	0	1
**T69D**	1	1	0
**T215S**	4	2	2
**T215E**	3	2	1
**T215A/S**	1	1	0
**T215D/E**	1	1	0
**T215T/S**	2	1	1
**T215L**	3	1	2
**T215D/N**	1	0	1
**T215T/A**	1	0	1
**E44D**	1	1	0

**Table 3:** Results of the Cox model stratifying patients for the presence of pre-treatment DRMs.

Variables	Univariable aHR (95% CI)	*p*-Value	Multivariable aHR (95% CI)	*p*-Value
**ART:**				
- **DTG, no DRMs**	1	Ref.	1	Ref.
- **DRV, no DRMs**	**1.96 (1.05–3.66)**	**0.035**	**2.33 (1.17–4.66)**	**0.016**
- **DTG, DRMs**	**6.21 (1.40–27.55)**	**0.016**	**17.27 (3.24–92.15)**	**0.001**
- **DRV, DRMs**	n.a.	n.a.	n.a.	n.a.
**HIV-1 subtype**				
- **B**	1	Ref.	1	Ref.
- **A**	1.35 (0.42–4.41)	0.616	1.35 (0.40–4.59)	0.630
- **C**	2.66 (0.94–7.53)	0.065	**3.10 (1.07–8.98)**	**0.037**
- **CRF02_AG**	1.75 (0.77–3.95)	0.181	1.68 (0.72–3.93)	0.233
- **CRF01_AE**	1.49 (0.20–10.92)	0.694	2.14 (0.27–16.99)	0.473
- **F**	0.99 (0.24–4.11)	0.986	1.04 (0.25–4.44)	0.955
- **G**	2.62 (0.93–7.42)	0.069	**4.23 (1.44–12.47)**	**0.009**
- **Other**	1.25 (0.30–5.19)	0.763	2.02 (0.41–10.07)	0.390
**HIV-RNA at baseline (copies/mL)**				
- **≤100.000**	1	Ref.	1	Ref.
- **100.001–500.000**	**3.45 (1.76–6.78)**	**<0.001**	**4.24 (2.07–12.97)**	**<0.001**
- **>500.000**	**4.93 (2.34–0.36)**	**<0.001**	**5.81 (2.60–2.97)**	**<0.001**
- **Unknown**	0.84 (0.27–2.56)	0.754	0.61 (0.14–2.60)	0.505
**CD4 cell count at baseline (cells/mmc)**				
- **≤200**	1	Ref.	1	Ref.
- **>200**	**0.57 (0.32–0.99)**	**0.047**	0.60 (0.33–1.08)	0.091
- **Unknown**	0.50 (0.21–1.21)	0.126	1.02 (0.33–3.19)	0.969
**Age (every 10 years more)**	0.98 (0.93–1.04)	0.506	1.01 (0.94–1.09)	0.752
**Time since HIV diagnosis**				
- **≤1 month**	1	Ref.	1	Ref.
- **>1 month**	**2.98 (1.26–7.03)**	**0.013**	**2.76 (1.17–6.49)**	**0.021**
- **Unknown**	**3.36 (1.44–7.87)**	**0.005**	**3.31 (1.42–7.70)**	**0.006**
**Gender**				
- **Female**	1	Ref.	1	Ref.
- **Male**	1.68 (0.76–3.71)	0.200	2.07 (0.90–4.74)	0.085
- **Unknown**	0.94 (0.12–7.62)	0.951	2.82 (0.22–35.89)	0.424
**CDC class C at baseline**				
- **No**	1	Ref.	1	Ref.
- **Yes**	0.36 (0.05–2.62)	0.314	0.15 (0.02–1.24)	0.079

**Table 4:** Results of the Cox model stratifying patients for viral subtypes.

Variables	Univariable aHR (95% CI)	*p*-Value	Multivariable aHR (95% CI)	*p*-Value
**ART:**				
- **DTG, B subtype**	1	Ref.	1	Ref.
- **DTG, A subtype**	4.07 (0.49–33.91)	0.194	7.83 (0.73–84.13)	0.089
- **DTG, C subtype**	3.93 (0.47–32.73)	0.205	**10.24 (1.12–93.55)**	**0.039**
- **DTG, CRF02-AG subtype**	2.08 (0.42–10.30)	0.370	1.95 (0.38–10.11)	0.425
- **DTG, CRF01-AE subtype**	**9.72 (1.15–81.99)**	**0.037**	**10.65 (1.18–96.04)**	**0.035**
- **DTG, F subtype**	2.74 (0.33–22.78)	0.351	2.37 (0.28–20.43)	0.431
- **DTG, G subtype**	4.09 (0.49–34.07)	0.192	4.75 (0.49–46.50)	0.180
- **DTG, other subtype**	3.66 (0.74–18.14)	0.113	4.57 (0.85–24.60)	0.077
- **DRV, B subtype**	**2.69 (1.11–6.53)**	**0.028**	**3.35 (1.33–8.44)**	**0.011**
- **DRV, A subtype**	2.41 (0.49–11.94)	0.283	3.09 (0.60–15.82)	0.176
- **DRV, C subtype**	**6.29 (1.57–25.17)**	**0.009**	**8.10 (1.90–34.48)**	**0.005**
- **DRV, CRF02-AG subtype**	**5.08 (1.55–6.65)**	**0.007**	**5.59 (1.62–19.24)**	**0.006**
- **DRV, CRF01-AE subtype**	n.a.	n.a.	n.a.	n.a.
- **DRV, F subtype**	1.62 (0.19–13.43)	0.657	2.23 (0.25–19.81)	0.472
- **DRV, G subtype**	**6.04 (1.51–24.16)**	**0.011**	**13.90 (3.30–58.57)**	**<0.001**
- **DRV, other subtype**	n.a.	n.a.	n.a.	n.a.
**DRMs (at least potential low-level resistance to one NRTI in the backbone versus none)**	3.08 (0.75–12.66)	0.119	11.02 (2.08–58.32)	**0.005**
**HIV-RNA at baseline (copies/mL)**				
- **≤100,000**	1	Ref.	1	Ref.
- **100,001–500,000**	**3.45 (1.76–6.78)**	**<0.001**	**3.96 (1.94–8.11)**	**<0.001**
- **>500,000**	**4.93 (2.34–0.36)**	**<0.001**	**5.41 (2.42–12.12)**	**<0.001**
- **Unknown**	0.84 (0.27–2.56)	0.754	0.45 (0.10–2.11)	0.313
**CD4 cell count at baseline (cells/mmc)**				
- **≤200**	1	Ref.	1	Ref.
- **>200**	**0.57 (0.32–0.99)**	**0.047**	0.62 (0.34–1.13)	0.116
- **Unknown**	0.50 (0.21–1.21)	0.126	1.09 (0.32–3.76)	0.889
**Age (every 10 years more)**	0.98 (0.93–1.04)	0.506	1.01 (0.93–1.09)	0.864
**Time since HIV diagnosis**				
- **≤1 month**	1	Ref.	1	Ref.
- **>1 month**	**2.98 (1.26–7.03)**	**0.013**	**3.02 (1.26–7.23)**	**0.013**
- **Unknown**	**3.36 (1.44–7.87)**	**0.005**	**3.75 (1.57–8.96)**	**0.003**
**Gender**				
- **Female**	1	Ref.	1	Ref.
- **Male**	1.68 (0.76–3.71)	0.200	2.08 (0.90–4.78)	0.086
- **Unknown**	0.94 (0.12–7.62)	0.951	2.33 (0.14–40.00)	0.561
**CDC class C at baseline**				
- **No**	1	Ref.	1	Ref.
- **Yes**	0.36 (0.05–2.62)	0.314	0.20 (0.03–1.50)	0.118

## Data Availability

The data presented in this study are available on request from the corresponding author. The data are not publicly available due to ethical restrictions.

## Supplementary Materials

The following supporting information can be downloaded at: https://www.mdpi.com/article/10.3390/v15030762/s1, Table S1: Pattern of pre-treatment resistance mutations associated with at least potential low-level resistance to one or both the NRTIs in the regimen.
