# Supplementary material for: Efficacy of Dolutegravir versus Darunavir in Antiretroviral First-Line Regimens According to Resistance Mutations and Viral Subtype
Source: Viruses. 2023 Mar 16;15(3):762. doi: 10.3390/v15030762 (PMC10059835; doi:10.3390/v15030762)
Supplement: Supplementary file 1 [file viruses-15-00762-s001.zip › viruses-2203734-supplementary.pdf]

Supplementary table S1. Pattern of pre-treatment resistance mutations associated with at least potential low-level resistance to one or both the NRTIs in the regimen.

Patient	Regimen	Pre-treatment DRM	Predicted level of resistance	Drug resistance mutation score	Virological failure
1	TFV/FTC+DTG	D67N/S, T69D, L210W, T215S,	Potential low-level resistance to TFV, Susceptible to FTC	TFV: 10<br>FTC: 0	No
2	TFV/FTC+DTG	M41L, V75M, L210W, T215A/S	Low-level resistance to TFV, Susceptible to FTC	TFV: 20<br>FTC: 0	No
3	TFV/FTC+DTG	M41L, T215C/F/I/L/R/S	Low-level resistance to TFV, Susceptible to FTC	TFV: 25<br>FTC: 5	Yes
4	TFV/FTC+DTG	D67N, K219Q	Potential low-level resistance to TFV, Susceptible to FTC	TFV: 10<br>FTC: 0	No
5	TFV/FTC+DTG	M184V	Susceptible to TFV, High-level resistance to FTC	TFV: -10<br>FTC: 60	Yes
6	ABC/3TC+DRV	M184V	Low-Level Resistance to ABC, High-level resistance to 3TC	ABC: 15<br>3TC: 60	No
7	ABC/3TC+DRV	M41L, D67N, M184V, L210W, K219E,	Intermediate resistance to ABC, High-level resistance to 3TC	ABC: 45<br>3TC: 60	No
8	TFV/FTC+DRV	D67N, K70R, M184V, K219Q	Low level resistance to TFV, High level resistance to FTC	TFV: 15<br>FTC: 70	No
9	TFV/FTC+DRV	K70K/T	Low-level resistance to TFV, Potential low-level resistance to FTC	TFV: 15<br>FTC: 10	No
